# Chromosome-Level Genome Assembly of a Fragrant *Japonica* Rice Cultivar ‘Changxianggeng 1813’ Provides Insights into Genomic Variations between Fragrant and Non-Fragrant *Japonica* Rice

**DOI:** 10.3390/ijms23179705

**Published:** 2022-08-26

**Authors:** Ruisen Lu, Jia Liu, Xuegang Wang, Zhao Song, Xiangdong Ji, Naiwei Li, Gang Ma, Xiaoqin Sun

**Affiliations:** 1 Institute of Botany, Jiangsu Province and Chinese Academy of Sciences, Nanjing 210014, China; 2 Changshu Agricultural Science Research Institute, Changshu 215500, China; 3 Guangdong Academy of Forestry, Guangzhou 510520, China

**Keywords:** *BADH2*, ‘Changxianggeng 1813’, fragrant rice, genome assembly, genomic variations, *japonica* cultivar

## Abstract

East Asia has an abundant resource of fragrant *japonica* rice that is gaining increasing interest among both consumers and producers. However, genomic resources and in particular complete genome sequences currently available for the breeding of fragrant *japonica* rice are still scarce. Here, integrating Nanopore long-read sequencing, Illumina short-read sequencing, and Hi-C methods, we presented a high-quality chromosome-level genome assembly (~378.78 Mb) for a new fragrant *japonica* cultivar ‘Changxianggeng 1813’, with 31,671 predicated protein-coding genes. Based on the annotated genome sequence, we demonstrated that it was the *badh2-E2* type of deletion (a 7-bp deletion in the second exon) that caused fragrance in ‘Changxianggeng 1813’. Comparative genomic analyses revealed that multiple gene families involved in the abiotic stress response were expanded in the ‘Changxianggeng 1813’ genome, which further supported the previous finding that no generalized loss of abiotic stress tolerance associated with the fragrance phenotype. Although the ‘Changxianggeng 1813’ genome showed high genomic synteny with the genome of the non-fragrant *japonica* rice cultivar Nipponbare, a total of 289,970 single nucleotide polymorphisms (SNPs), 96,093 small insertion-deletion polymorphisms (InDels), and 8690 large structure variants (SVs, >1000 bp) were identified between them. Together, these genomic resources will be valuable for elucidating the mechanisms underlying economically important traits and have wide-ranging implications for genomics-assisted breeding in fragrant *japonica* rice.

## 1. Introduction

Fragrant rice (*Oryza sativa* L.), well-known for its pleasant and subtle aroma, is widely preferred among rice consumers and fetches a higher price than non-fragrant rice in both domestic and international markets [1,2]. At present, Basmati rice from India and Pakistan and Jasmine rice from Thailand are the two most popular fragrant rice cultivars in the world [3,4]. It is, however, noteworthy that both of these two fragrant rice cultivars belong to the *indica* subspecies, with fluffy and dry cooked rice, while consumers from East Asia, including China, Japan, and Korea tend to prefer *japonica* rice that becomes sticky and soft when cooked [4]. Although East Asia has diverse and rich germplasm resources of fragrant *japonica* rice, none of them have been fully commercially utilized [5]. Thus, breeding and cultivation of fragrant *japonica* rice has become one of the most important jobs in modern rice breeding projects, especially in East Asia [6].

Hundreds of volatile compounds have been detected in fragrant rice, but the key compound responsible for the characteristic fragrance is 2-acetyl-1-pyrroline (2AP) [2,7]. Investigations into the genetic basis of rice fragrance have demonstrated that the fragrance phenotype is largely controlled by a recessive *betaine aldehyde dehydrogenase 2* (*BADH2*) gene, which comprises 15 exons and 14 introns with approximately 7 kilobase pairs in length [7,8]. The dominant *BADH2* gene encoding the active BADH2 catalyzes the oxidation of γ-aminobutyraldehyde (AB-ald, a 2AP precursor), while the recessive *BADH2* gene encoding the inactive BADH2 results in the accumulation of both AB-ald and its cyclic form Δ^1^pyrroline, and finally acetylates 2AP through enzymatic or non-enzymatic reactions [7,9,10,11]. To date, multiple types of loss-of-function mutations in the *BADH2* gene responsible for rice fragrance have been reported, e.g., an 8-bp deletion and three single nucleotide polymorphisms (SNPs) in the seventh exon (designated as *badh2-E7* or *badh2.1*), a 7 bp deletion in the second exon (*badh2-E2* or *badh2.2*), and an 803 bp deletion between the fourth and fifth exons (*badh2-E4/5*) [6,7,12,13,14]. Based on the above information, functional molecular markers have also been developed for various SNPs and small insertion-deletion polymorphisms (InDels) on different exons of *BADH2*, improving the efficiency of selection and breeding of fragrant rice, e.g., [6,13]. However, these molecular markers were usually developed based on old conventional fragrant rice varieties, most of which have relatively low yields and demonstrate inferior agronomic performance, such as weak disease resistance and low tolerance to climatic stresses [3]. Thus, excluding inferior agronomic traits has become a major challenge during introgression of fragrance alleles from old conventional fragrant rice cultivars into modern rice cultivars [3].

The new fragrant *japonica* rice cultivar ‘Changxianggeng 1813’ (2AP content: ~310 ug/kg, unpublished data), derived from a cross between ‘93-63/wuyungeng 20’ and ‘wuyungeng 31’, was developed by the Changshu Institute of Agricultural Sciences (Changshu, Jiangsu, China) and licensed for release in Jiangsu Province, China in 2020 [15]. In contrast to old conventional fragrant *japonica* rice cultivars, ‘Changxianggeng 1813’ shows high resistances to lodging and blast, with both high yield and good quality [15], which is not only suitable for being widely planted in the South Yangtze River regions, but also could be used as a parental line to develop new fragrant *japonica* rice cultivars. Therefore, the construction of a high-quality genome of ‘Changxianggeng 1813’ is essential for further improvement of this cultivar or its progenies, as well as accelerating the process of fragrant *japonica* rice breeding, by providing genomic resources that could be directly applied to fragrant *japonica* rice cultivars.

With the rapid progress in next-generation sequencing technologies, unprecedented amounts of genomic data for wild and cultivated rice are currently available, providing important resources for investigation of the genetic basis behind rice domestication and improvement [16,17,18,19,20,21,22,23,24,25,26,27,28,29]. Within cultivated rice, however, genome assemblies for most cultivars were based on short-read sequencing data, which often showed higher levels of incompleteness than those generated from long-read sequences, e.g., [16,30,31,32]. Moreover, the information from highly polymorphic regions, especially for large structural variations (SVs), would often be inevitably lost by direct mapping of short sequencing reads onto a single reference genome (typically, *O. sativa japonica* Nipponbare) [23,33]. Thus, high-quality, chromosome-level genome assemblies for different rice cultivars are still needed to comprehensively capture the genomic variations in rice.

In this study, we generated a high-quality, chromosome-level genome sequence of the fragrant *japonica* rice cultivar ‘Changxianggeng 1813’, based on Oxford Nanopore, Illumina, and Hi-C sequencing technologies. Then, we aligned the *BADH2* gene in ‘Changxianggeng 1813’ to previously described *BADH2* haplotypes to verify the presence/absence of the mutations associated with fragrance and determine their phylogenetic relationships. We also carried out comparative genomic analyses to provide insights into the evolution and adaptation of this cultivar. Finally, we performed a pairwise genome comparison between the fragrant *japonica* cultivar ‘Changxianggeng 1813’ and the non-fragrant *japonica* cultivar Nipponbare to identify genomic variations (SNPs, InDels, SVs). Of note, this is the first high-quality de novo assembly genome sequence for fragrant *japonica* rice published to date, and is expected to have a lasting direct impact on molecular breeding and improvement of fragrant *japonica* rice.

## 2. Results and Discussion

### 2.1. Genome Sequencing and De Novo Assembly

With the rapid development of genome sequencing methods, long-read sequencing technologies such as Oxford Nanopore Technology and Pacific Biosciences combined with Illumina short-read sequencing and chromosome conformation capture (Hi-C) technologies have become a common standard protocol to generate high-quality assemblies of plant genomes [34,35,36]. In this study, the genome of ‘Changxianggeng 1813’ was sequenced and de novo assembled by a hybrid strategy combining Oxford Nanopore, Illumina, and Hi-C technologies. A total of ~51.59 Gb Nanopore long reads, ~28.21 Gb Illumina short reads, and ~41.45 Gb Hi-C reads were generated, respectively, after filtering (Table S1). Using *k*-mer analysis with Illumina clean reads, the genome size of ‘Changxianggeng 1813’ was estimated to be approximately 394.39 Mb, with a heterozygosity rate of 0.08% (Table S2).

**Figure 1:**
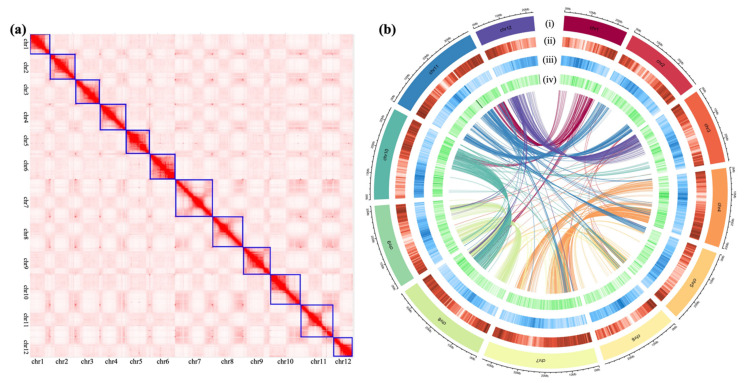
Basic characteristics of the ‘Changxianggeng 1813’ genome. (**a**) Genome-wide Hi-C heat map of the ‘Changxianggeng 1813’ genome showing chromatin interactions among the 12 chromosomes. Darker red color indicates higher contact probability. The blue boxes show the location of the chromosomes. (**b**) Circos plot of the multidimensional topography of the 12 chromosomes in the ‘Changxianggeng 1813’ genome. Concentric circles, from outermost to innermost, show (i) the chromosome, (ii) gene density, (iii) percentage of repeats, and (iv) GC content. The three metrics were calculated in 500 kb sliding windows. In the innermost circle, each line shows the syntenic relationship between different chromosomes, indicating the existence of large episodic duplications derived from the ancient whole-genome duplication in rice.

The ‘Changxianggeng 1813’ genome was preliminarily assembled based on Nanopore long reads, followed by two rounds of assembly corrections using both of Nanopore and Illumina sequencing data, which produced an assembled genome (scaffold level) with a total length of ~378.78 Mb, a GC content of 43.55%, and a surprisingly long scaffold N50 of 29.83 Mb (Table 1). Despite the super-long scaffolds generated, Hi-C data were employed to further improve assembly contiguity and obtain a high-quality reference genome of ‘Changxianggeng 1813’. Approximately 62.20 million valid interaction pairs (~18.65 Gb Hi-C data), accounting for 82.55% of the unique mapped read pairs, were used for the Hi-C assembly. Consequently, all ~378.78 Mb (100%) data in 20 scaffolds were anchored and orientated onto 12 chromosomes by agglomerative hierarchical clustering, with their lengths ranging from 22.66 to 43.60 Mb (Figure 1a,b; Table S3). The 12 chromosomes could be distinguished obviously, and the near-diagonal interaction signals were considerably stronger than that of other positions within each chromosome, which illustrated that Hi-C scaffolding was reliable and robust (Figure 1a).

The accuracy and completeness of the genome assembly were first assessed by mapping the Illumina reads back to the reference genome, which revealed a mapping efficiency of 99.11% (Table 1). Furthermore, 1552 (96.16%) of 1614 conserved BUSCO (Benchmarking Universal Single-Copy Orthologs) genes, including 1514 (93.8%) complete and single-copy BUSCOs and 38 (2.4%) complete and duplicated BUSCOs (Table 1 and Table S4), were completely recalled in our assembly. Taken together, these results implied that the genome assembly of ‘Changxianggeng 1813’ was performed well and in high completeness. In a word, the assembled genome of ‘Changxianggeng 1813’ was at the chromosomal level, with a longer scaffold N50 length than in most de novo assemblies of *Oryza* genomes e.g., [16,31,37,38], which provides good quality, high-resolution resources for associating traits of interest with genetic variations and identifying the genes controlling those important economical traits in fragrant *japonica* rice.

### 2.2. Genome Annotation

Repetitive sequences constitute large proportions of plant genomes and often play key roles in plant genome evolution due to their roles in both genome size variation and functional adaption [39,40]. Using a combination of homology-based and de novo approaches, about 50.52% of the ‘Changxianggeng 1813’ genome was identified as transposable elements (TEs; Table S5). Of these TEs, DNA transposons were the most abundant, occupying 24.47% of the genome, followed by long terminal repeats (LTRs; 24.15%), while long interspersed nuclear elements (LINEs) and short interspersed nuclear elements (SINEs) accounted for 1.78% and 0.12%, respectively (Table S5). Additionally, approximately 0.97% of the ‘Changxianggeng 1813’ genome was identified as tandem repeats (Table S5). Indeed, among various types of repetitive sequences, LTRs are one of the most important contributors to the genome size variation across the *Oryza* genus [41,42]. It was thus speculated that the genome size of ‘Changxianggeng 1813’ (~379 Mb), nearly half of that in *O. granulate* (~777 Mb), is largely due to the differences of the proportion of LTRs between them (24.15% for ‘Changxianggeng 1813’ and 59.33% for *O. granulate*) [24].

A total of 32,165 protein-coding genes were predicted by integrating protein-based homology, de novo and transcriptome-based prediction approaches, with average gene and coding sequence lengths of 4244 and 1224 bp, respectively, and an average of 4.62 exons per gene (Table 2). Among these protein-coding genes, 98.46% (31,671) could be annotated by at least one of the six functional databases employed, including Uniprot, Pfam, GO (Gene Ontology), KEGG (Kyoto Encyclopedia of Genes and Genomes), and NR (Non-redundant) (Table S6). In addition, 3524 RNAs were identified as potential noncoding RNAs, including 1756 microRNAs (miRNAs), 715 transfer RNAs (tRNAs), 322 ribosomal RNAs (rRNAs), and 731 small nuclear RNAs (snRNAs) (Table S7).

### 2.3. Characterization and Evolutionary Analysis of BADH2 Gene

Since ‘Changxianggeng 1813’ has been identified to be a fragrant rice (2AP content: ~310 ug/kg, unpublished data), we checked whether it indeed carries a recessive *BADH2* gene and investigated its allelic variation. By comparing to the non-fragrant rice cultivar Nipponbare, a 7 bp deletion (5′-CGGGCGC-3′) in the second exon was observed at the *BADH2* allele (*badh2-E2*), which generated a premature stop codon that disabled the BADH2 enzyme (Figure S1a), thereby promoting the accumulation of 2AP in ‘Changxianggeng 1813’. The *badh2-E2* allele carried by ‘Changxianggeng 1813’ was consistent with that in a number of Chinese fragrant *japonica* rice cultivars, e.g., ‘Wuxiang 9915’, ‘Xiangjing 111’, ‘Zhenxiangjing 5’, suggesting that this allele is common by descent in Chinese fragrant rice cultivars [8,13]. Phylogenetic analysis of *BADH2* haplotype data (Figure S1b) further showed that the *badh2-E2* allele in ‘Changxianggeng 1813’ clustered together with a previously identified haplotype sequence endemic to two cultivated *japonica* rice (see [2] for full details). Taken together, these findings provided additional support for the previous studies indicating that *badh2-E2* may arise and become fixed in the *japonica* gene pool [7,8,13]. However, it is worth noting that the sample size analyzed to date is still inadequate to comprehensively detect the origin and evolution of *badh2-E2* in fragrant rice.

### 2.4. Genome Synteny

**Figure 2:**
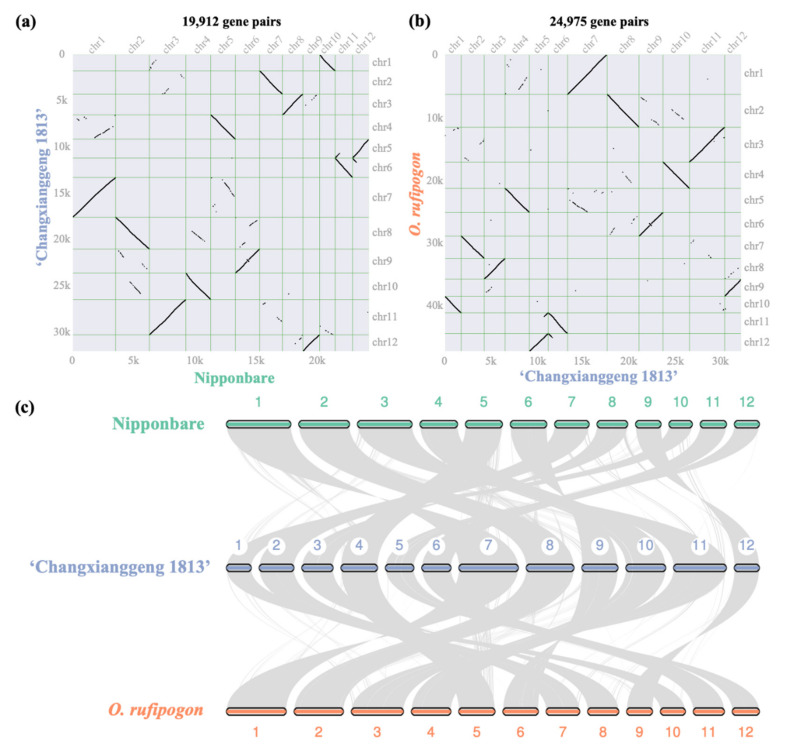
Chromosome synteny between the fragrant *japonica* cultivar ‘Changxianggeng 1813’ and its close relatives, i.e., the non-fragrant *japonica* cultivar Nipponbare and the common wild rice *O. rufipogon*. (**a**,**b**) Syntenic dot plots for intergenomic comparisons of (**a**) ‘Changxianggeng 1813’ vs. Nipponbare, and (**b**) ‘Changxianggeng 1813’ vs. *O. rufipogon*. (**c**) Macrosyntenic relationship pattern between ‘Changxianggeng 1813’ and its two close relatives (Nipponbare and *O. rufipogon*).

Comparisons of genome synteny within and between species have provided a framework to reveal evolutionary processes that lead to diversity of genome structure and function in many lineages [43]. Nowadays, genome synteny analysis has become an integral part of comparative genomics for almost every new published genome. Using the MCScan toolkit, a total of 19,912 and 24,975 gene pairs were identified in the intergenomic comparisons of the fragrant cultivar ‘Changxianggeng 1813’ vs. the non-fragrant cultivar Nipponbare (Figure 2a), and ‘Changxianggeng 1813’ vs. the common wild rice *O. rufipogon* (Figure 2b), respectively. In general, extremely high degrees of collinearity were observed in these two comparisons; each chromosome of ‘Changxianggeng 1813’ corresponded to one chromosome of Nipponbare and *O. rufipogon*, respectively, although some interchromosomal rearrangement events were detected (Figure 2c). It was also found that there were fewer scattered points in the comparison of ‘Changxianggeng 1813’ vs. Nipponbare, than in ‘Changxianggeng 1813’ vs. *O. rufipogon* (Figure 2a,b), suggesting a close relationship between ‘Changxianggeng 1813’ and Nipponbare.

### 2.5. Gene Family Evolution and Phylogenetic Relationships

**Figure 3:**
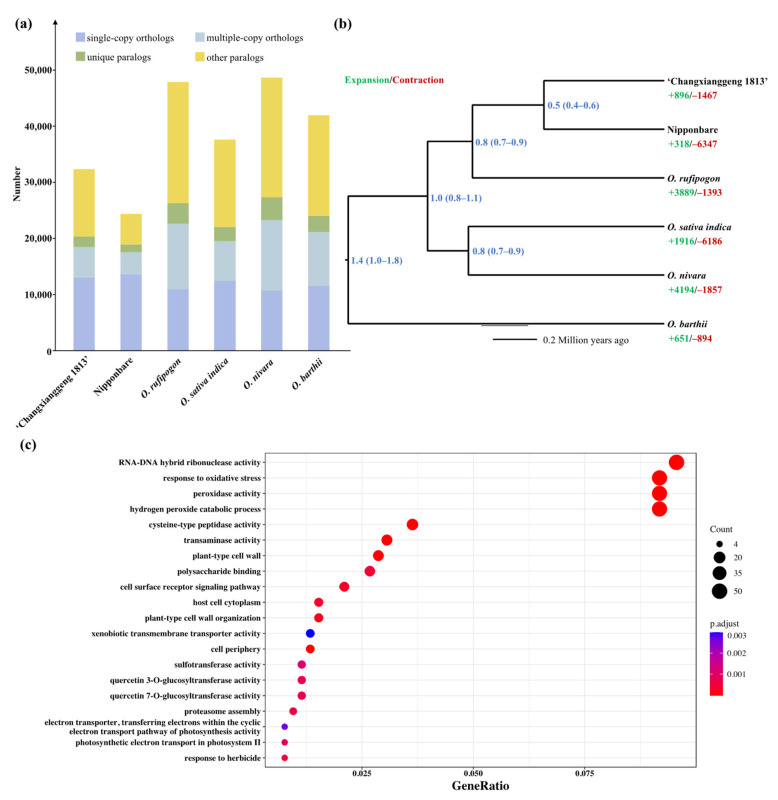
(**a**) Comparison of copy numbers in gene clusters residing in the genomes of ‘Changxianggeng 1813’ and five other members of *Oryza*. (**b**) Phylogenetic tree inferred from single-copy orthogroups. Numbers near each node refer to divergence times (in million years ago, Ma). Bootstrap values are all 100. Numbers marked in green and red represent gene family expansions and contractions, respectively. (**c**) Visualization of results from GO enrichment analysis of significantly expanded gene families in ‘Changxianggeng 1813’. The top 20 GO terms were selected for display after using the Benjamini–Hochberg multiple test correction for *p*-value adjustment (adjusted *p*-value < 0.01).

Of the 32,165 protein-coding genes identified in the ‘Changxianggeng 1813’ genome, 11,413 were classified as single-copy orthologs, 9531 as multiple-copy orthologs, 2899 as unique paralogs, and 17,837 as other paralogs (Figure 3a). All the 32,165 protein-coding genes were clustered into 26,658 gene families, of which 1576 (5.91%) were unique in the ‘Changxianggeng 1813’ genome (Table S8). A total of 7658 single-copy orthologous genes shared among the six *Oryza* genomes were identified and used for phylogenetic analysis. Phylogenetic analysis strongly supported that the fragrant cultivar ‘Changxianggeng 1813’ and the non-fragrant cultivar Nipponbare, both of which belong to the *japonica* subspecies, were sister to each other, and jointly sister to the common wild rice *O. rufipogon* (Figure 3b). The divergence time of ‘Changxianggeng 1813’ and Nipponbare was estimated to be 0.5 (0.4–0.6) million years ago (Ma; Figure 3b), unambiguously older than the date of domestication of the rice (10,000 years ago). One possible explanation for this is that the divergence between these two cultivars from two different subpopulations (temperate *japonica* and *aromatic*) is in part due to differentiation of their ancestral populations in different locations and/or at different times. Furthermore, although our estimated divergence time is slightly older than the date for *japonica* and *indica* (about 0.44 Ma), this estimate conformed generally with the previous findings that (i) genomic variation in the rice is deeply partitioned and that divergent haplotypes can be readily associated with major varietal groups and subpopulations, and (ii) rice domestication proceeded from multiple predifferentiated ancestral pools much earlier than the beginning of agriculture in Asia [37,44].

Gene family expansion and contraction are generally considered important evolutionary mechanisms that contribute to evolutionary adaption to the environment [45,46]. To reveal gene family expansion and contraction related to environmental stress in ‘Changxianggeng 1813’, we undertook a computational analysis of gene family sizes among different members of *Oryza*. Our results indicated that 896 gene families in ‘Changxianggeng 1813’ genome underwent expansion, while 1467 genes families underwent contraction (Figure 3b). Functional enrichment analysis of expanded gene families revealed 25 GO terms that were significantly enriched (*p*.adjust < 0.01). The expanded gene families were mainly enriched in genes associated with RNA-DNA hybrid ribonuclease activity (GO:0004523, *p*.adjust = 2.6 × 10^−30^), hydrogen peroxide catabolic process (GO:0042744, *p*.adjust = 1.71 × 10^−37^), peroxidase activity (GO:0004601, *p*.adjust = 5.05 × 10^−35^), and response to oxidative stress (GO:0006979, *p*.adjust = 8.24 × 10^−32^) (Figure 3c). It needs to be emphasized here that oxidative stress is regarded as a major damaging factor in plants exposed to a variety of abiotic stresses [47]. Thus, these expanded oxidative stress response genes may have a role in conferring enhanced stress tolerance to ‘Changxianggeng 1813’ during periods of rapid climate change. This result also supported the previous findings that *BADH2* does not play a role in abiotic stress tolerance in rice, and no generalized loss of abiotic stress tolerance associated with the fragrance phenotype [48].

### 2.6. Genomic Variations between ‘Changxianggeng 1813’ and Nipponbare

**Figure 4:**
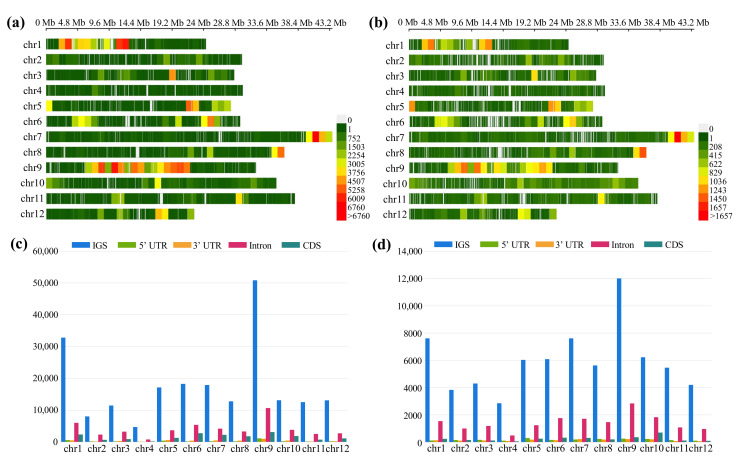
(**a**,**b**) Distribution patterns of SNPs (**a**) and InDels (**b**) across the ‘Changxianggeng 1813’ genome by comparing to the Nipponbare genome. (**c**,**d**) The distribution of (**c**) SNPs and (**d**) InDels in different genomic regions, including intergenic spacer regions (IGS), 5′ untranslated regions (UTR), 3′ UTR, intron and protein coding regions (CDS).

Since large-scale genome sequencing has been undertaken in rice, a substantial number of genetic variations, such as single nucleotide polymorphisms (SNPs) and small insertion-deletion polymorphisms (InDels), have become available across the rice genome, e.g., [26,49]. However, few recent studies have been concentrated on fragrant *japonica* rice, resulting in a severe lack of knowledge for valuable fragrant *japonica* rice, especially in East Asia. Although the genome assembly of the fragrant *japonica* cultivar ‘Changxianggeng 1813’ very closely matched the genome of non-fragrant *japonica* cultivar Nipponbare (Figure 2a,c), a total of 289,970 SNPs and 96,093 InDels were identified in the ‘Changxianggeng 1813’ genome when compared to the Nipponbare genome, with an average density of 0.76 SNPs and 0.25 InDels per kb, respectively (Figure 4; Tables S9 and S10). The number of SNPs and InDels per 1 Mb varied considerably across each chromosome. In particular, chromosome 9 had the highest density of both SNPs (208.3 Mb^−1^) and InDels (49.0 Mb^−1^), while chromosome 4 had the lowest SNP (19.6 Mb^−1^) and Indel (1.2 Mb^−1^) densities (Figure 4a,b; Tables S9 and S10). The distribution of SNPs and InDels was also uneven within a chromosome. For example, on chromosome 1, SNPs and InDels were dense from 11.9 to 12.7 Mb, but sparse from the regions of 9.8–10.5 and 17.6–20.0 Mb (Figure 4a,b). The distributions of SNPs and InDels were positively correlated, and both were more abundant in intergenic spacer (IGS) regions. More specifically, about 67.12% (13,142/19,580, chromosome 10) to 80.07% (4728/5905, chromosome 4) of SNPs and 67.62% (6233/9217, chromosome 10) to 79.20% (2856/3606, chromosome 4) of InDels were located in the IGS regions (Figure 4c,d; Tables S9 and S10). The distributions of the SNPs and InDels in the genomic regions were also examined, which indicated that most of them were in the introns (SNPs: 57.36% on chromosome 8 to 70.20% on chromosome 11; InDels: 61.53% on chromosome 10 to 76.89% on chromosome 12), while 5′ UTRs, 3′ UTRs, and CDS contained only a small fraction (Figure 4c,d; Tables S9 and S10). The information described here can be exploited in future studies to provide novel perspectives on genetics and breeding of fragrant *japonica* rice.

**Figure 5:**
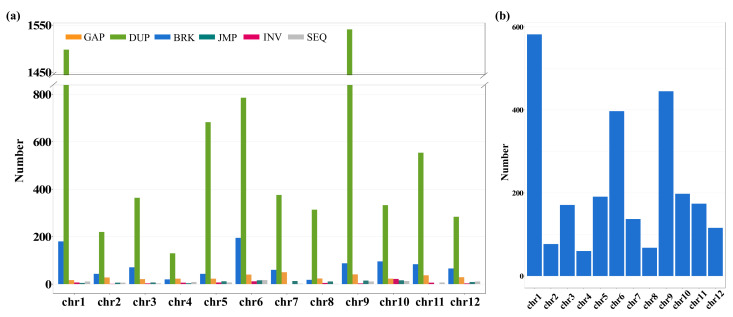
(**a**) SV types and numbers across 12 chromosomes of the ‘Changxianggeng 1813’ genome. (**b**) Total counts of SVs overlapping genes for each chromosome in the ‘Changxianggeng 1813’ genome. GAP, gap between two mutually consistent alignments; DUP, inserted duplication; BRK, other inserted sequence; JMP, rearrangement; INV, rearrangement with inversion; SEQ, rearrangement with another sequence.

It is also noteworthy that SNPs and small InDels do not capture all the meaningful genomic variations that underlie crop improvement, and that structure variants (SVs) also play an important role in plant evolution and agriculture [50,51]. SVs typically defined as genomic variations that involve segments of DNA larger than 1 kb in length, hence detecting SVs with short-read sequencing is a challenging problem, leaving the vast majority of SVs poorly resolved in rice [26,33]. Nowadays, the recent development of high-throughput Oxford Nanopore long-read sequencing has enabled us to take a broad survey on previously hidden SVs in rice genomes [16]. In this study, establishing a high-quality de novo genome assembly for ‘Changxianggeng 1813’ allowed us to resolve large SVs between fragrant and non-fragrant *japonica* rice. A total of 8690 large SVs were identified between the genomes of ‘Changxianggeng 1813’ and Nipponbare through direct genome comparison (Figure 5a, Table S11). Of these SVs, the dominant type was DUP (gap between two mutually consistent alignments), accounting for 81.51% (7083/8650) of all identified SVs, followed by BRK (other inserted sequence) (11.09%, 964/8650) and GAP (gap between two mutually consistent alignments) (4.10%, 356/8650), while the JMP (rearrangement) (1.31%, 114/8650), SEQ (rearrangement with another sequence) (1.13%, 98/8650), and INV (rearrangement with inversion) (0.86%, 75/8650) were least abundant (Figure 5a; Table S11).

The total number of SVs detected also varied across different chromosomes. To be specific, the highest number of SVs (Total: 1718; GAP: 17, DUP: 1598, BRK: 180, JMP: 5, INV: 7, SEQ: 11) was observed on chromosome 1, while chromosome 4 had the lowest number of SVs (Total: 192; GAP: 23, DUP: 130, BRK: 20, JMP: 4, INV: 6, SEQ: 9) (Figure 5a, Table S11). Because SVs overlapping genes can impact gene functions and expression, and those in noncoding genes have a disproportionate impact on gene expression of nearby genes [51,52], we examined the distributions of SVs in different genomic regions. Our results indicated that a majority (~70%) of SVs located in noncoding regions, notably higher than the proportion (~30%) in gene regions (Figure 5b, Table S11). As expected, SVs overlapping genes were also distributed unevenly on each chromosome, ranging from 60 SVs on chromosome 4 to 582 SVs on chromosome 1 (Figure 5b, Table S11). This result suggested that some regions might be conserved and share a common ancestral gene pool between the two *japonica* cultivars (‘Changxianggeng 1813’ and Nipponbare).

## 3. Materials and Methods

### 3.1. Plant Materials and DNA Extraction

Genomic DNA was extracted from fresh leaves of 15-day-old seedlings of the fragrant *japonica* cultivar ‘Changxianggeng 1813’ using the DNAsecure Plant Kit (Tiangen Biotech, Beijing, China) according to the manufacturer’s protocol. The quality and integrity of the DNA products were assessed using agarose gel electrophoresis, NanoDrop spectrophotometry (NanoDrop Technologies, Wilmington, DE, USA), and Qubit fluorometry (Thermo Fisher Scientific, Waltham, MA, USA). The genomic DNA that met the quality and quantity standards was used to construct Illumina and Nanopore libraries.

### 3.2. Genome and Transcriptome Sequencing

For Illumina sequencing, a short-insert (350 bp) genomic library was performed using the NEBNext Ultra DNA Library Prep Kit (New England Biolabs, Beverly, MA, USA), and sequenced on the Illumina NovaSeq 6000 platform using a paired-end sequencing strategy. To reduce the effect of sequencing errors, we discarded those reads that met either of the following criteria: (i) reads with adapters; (ii) reads having more than 50% bases with Phred quality < 5; (iii) reads with N bases more than 5%; and (iv) PCR duplicated reads. All the obtained clean reads were prepared to carry out genome size estimation, genome assembly correction and evaluation.

For Nanopore sequencing, approximately 10 μg of genomic DNA was size-selected (10–50 kb) with the BluePippin System (Sage Science, Beverly, MA, USA), and then the DNA was subjected to a 30 µL end-repair/dA-tailing reaction using the NEBNext Ultra End Repair/dA-Tailing module (New England Biolabs, Beverly, MA, USA). The sequencing adaptors were further ligated using the Ligation Sequencing Kit SQK-LSK109 (Oxford Nanopore Technologies, Oxford, UK) based on the manufacturer’s instructions. After purifying using Ampure XP beads and the ABB wash buffer (Oxford Nanopore Technologies), the resulting library was sequenced on R9.4 flow cells using the PromethION DNA sequencer (Oxford Nanopore Technologies). Raw signal data in fast5 format was subsequently base called using Guppy v.2.3.5 (Oxford Nanopore Technologies) with default parameters, and the reads with the mean_qscore_template <7 were filtered.

For chromatin conformation capture (Hi-C) sequencing, fresh leaves from the same ‘Changxianggeng 1813’ plant that were used for Illumina and Nanopore sequencing were collected. A Hi-C library was created in a similar manner to that described by Lieberman-Aiden et al. [53]. Briefly, chromatin was first fixed in 1% final concentration of formaldehyde, and the extracted fixed chromatin was digested using the restriction enzyme DpnII. The 5′ overhangs were then filled in with biotinylated nucleotides, and free blunt ends were ligated. After ligation, cross-links were reversed, and the DNA was purified from the protein. Purified DNA was further filtered to remove unligated but biotin-labeled fragments and subjected to selection for fragments with lengths between 300 and 700 bp. The quality of the purified library was evaluated with an Agilent 2100 instrument, a Qubit fluorometer (Thermo Fisher Scientific, Waltham, MA, USA), and quantitative PCR (qPCR). Finally, the qualified library was sequenced on an Illumina HiSeq X Ten platform with the layout of pair-ended 150 bp reads.

For transcriptome sequencing (RNA-Seq), the best-quality RNA samples of each tissue (root, branch, leaf, and panicle) were mixed together to build a Nanopore sequencing library using the Ligation Sequencing Kit (SQK-LSK109, Oxford Nanopore Technologies) by following the manufacturer’s protocol. The cDNA library was added to FLO-MIN109 flow cells and sequenced on the Nanopore PromethION platform. Raw reads were filtered with the following settings: minimum average read quality score 7 and minimum read length 500 bp. Ribosomal RNA was discarded by searching against the Silva rRNA database (https://www.arb-silva.de, accessed on 27 August 2020). The RNA-Seq data were used to improve the annotation of ‘Changxianggeng 1813’. All library construction, sequencing, and data filtering were conducted in Wuhan Benagen Tech Solutions Company Limited, Wuhan, China.

### 3.3. Genome Size Estimation and Genome Assembly

All Illumina clean reads were used for the estimation of genome size and heterozygosity with *k*-mer analysis. The data were run through Jellyfish v.2.3.0 [54] to generate *k*-mer frequency distribution, with a *k*-mer size of 19. Genome size was estimated by the commonly used formula: genome size = *k*-mer_number/*k*-mer_depth, where *k*-mer_number is the total number of *k*-mers, and *k*-mer_depth is the main peak of *k*-mer frequency.

NextDenovo v.2.4.0 (https://github.com/Nextomics/NextDenovo, accessed on 27 December 2020) was applied to de novo assembly of ‘Changxianggeng 1813’ genome using nanopore long reads. Briefly, the NextCorrect module was employed to correct raw reads and extract consensus sequences, and then the NextGraph module was used to assemble the draft genome. To improve the accuracy of the draft genome, we used Racon v.1.4.11 [55] and Pilon v.1.23 [56] to polish the assembly for two rounds, respectively, based on the corrected nanopore long reads and the cleaned Illumina short reads. After these two-step polishing strategies, the scaffold-level genome assembly was generated. To further anchor the genome assembly to the chromosome level, HiCUP v.0.6.17 [57] was used to produce cleaned mapped data accompanied with QC reports. Only uniquely aligned read pairs with mapping quality >20 were retained and utilized to cluster, order, and orient the assembly scaffolds onto chromosomes by LACHESIS software [58].

### 3.4. Quality Assessment of Genome Assembly

To evaluate the accuracy and completeness of the genome assembly, Illumina reads were mapped back to the reference genome using BWA-MEM v.0.7.17 [59] and assessed by their depth of coverage. Furthermore, BUSCO (Benchmarking Universal Single-Copy Orthologs) v.4.1.4 [60], with the database embryophyta_odb10, was employed to assess the completeness of the genome assembly.

### 3.5. Genome Annotation

The genome of ‘Changxianggeng 1813’ was annotated at three independent dimensions: (i) repetitive elements, (ii) protein-coding genes, and (iii) noncoding RNAs. For repetitive element annotation, transposable elements (TEs) in the ‘Changxianggeng 1813’ genome were identified using a hybrid strategy combining homology-based searching in known repeat database and de novo prediction. RepeatMasker v.4.0.6 [61] was used to identify TEs against both the RepBase database of known TEs [62], and a de novo repeat library constructed by RepeatModeler v.1.0.11 (http://www.repeatmasker.org/RepeatModeler/, accessed on 21 October 2017). TEs identified from both homology-based and de novo approaches were further filtered for redundant sequences and merged into a non-redundant repeat library by CD-HIT [63]. In addition, tandem repeats including microsatellites (SSRs) were identified in the reference genome of ‘Changxianggeng 1813’ using Tandem Repeat Finder (TRF) v.4.0.9 [64].

For protein-coding genes prediction, three different methods, including homology-based, de novo, and transcriptome-based methods were unitedly conducted. Frist, Exonerate v.2.4.0 [65] was used for the homology-based prediction, based on protein sequences of *O. brachyantha*, *O. sativa japonica* Nipponbare, *Aegilops tauschii*, and *Panicum hallii* retrieved from NCBI (http://www.ncbi.nlm.nih.gov, accessed on 28 June 2021). Then, Augustus v.3.3.2 [66] and GlimmerHMM v.3.0.4 [67] were applied for the de novo prediction, with default parameters. Next, TransDecoder v.5.1.0 (https://github.com/TransDecoder/TransDecoder/wiki, accessed on 28 March 2018) was employed to identify the potential coding regions, based on the assembled transcripts using Stringtie v.2.1.1 [68]. Finally, EvidenceModeler v.1.1.1 [69] was used to integrate the prediction results obtained through the above three methods to generate the final gene set of ‘Changxianggeng 1813’. Functional gene annotation was performed by aligning the protein sequences against Uniprot, Pfam, GO (Gene Ontology), KEGG (Kyoto Encyclopedia of Genes and Genomes), and NR (Non-redundant) databases, with an E-value threshold of 1 × 10^−5^. Furthermore, InterProScan v.5.33 [70] was used to annotate the motifs and domains by searching against the InterPro and Pfam databases. These results were further integrated to produce the final genes set.

For noncoding RNA prediction, transfer RNAs (tRNAs) and ribosomal RNAs (rRNAs) were predicted using tRNAscan-SE v.1.23 [71] and RNAmmer v.1.2 [72], respectively. Other types of noncoding RNAs, including small nuclear RNAs (snRNAs) and microRNAs (miRNAs) were identified by using Infernal v.1.1.2 [73] based on the Rfam database [74].

### 3.6. Characterization and Evolutionary Analysis of BADH2 Gene

The *BADH2* gene sequence for ‘Changxianggeng 1813’ was extracted from its genome sequence according to annotation files and then compared to that of the non-fragrant cultivar Nipponbare using the MAFFT multiple sequence alignment program [75], to verify the presence/absence of the mutations associated with fragrance in ‘Changxianggeng 1813’. The *BADH2* protein-coding sequence for ‘Changxianggeng 1813’, was further combined with previously published 38 haplotypes in the *BADH2* coding region for phylogenetic analysis [2]. All 39 *BADH2* coding sequences were aligned using ClustalW [76], and the resulting alignment was used for Neighbor-Joining (NJ) phylogenetic tree construction using MEGA v.11.0.11 [77], with 1000 bootstrap replicates.

### 3.7. Genome Synteny and Collinearity Analysis

To identify chromosome structural changes between ‘Changxianggeng 1813’ and its two close relatives, i.e., the non-fragrant *japonica* rice cultivar Nipponbare and the common wild rice *O. rufipogon*, genome syntenic blocks were identified using the Python version of MCscan incorporated in jcvi (https://github.com/tanghaibao/jcvi/wiki/MCscan-(Python-version), accessed on 16 June 2020), with default parameters. In brief, all-against-all LAST [78] was performed, and the LAST hits with a distance cutoff of ten genes and at least five syntenic genes per block were chained. Dot plots for pairwise synteny, and macrosyntenic patterns were generated using the commands ‘python-m jcvi.graphics.dotplot’ and ‘python-m jcvi.graphics.karyotype’, respectively.

### 3.8. Gene Family and Phylogenetic Analysis

OrthoMCL v.2.0.9 [79] was used to identify gene family clusters in the genomes of ‘Changxianggeng 1813’ and five other members of *Oryza*, including the non-fragrant *japonica* cultivar Nipponbare, the *indica* subspecies, and three wild species (*O. rufipogon*, *O. nivara*, and *O. barthii*). Low-quality protein sequences from these six *Oryza* genomes were firstly filtered, based on default parameters in OrthoMCL. Then, an all-versus-all BLASTP search was conducted for all remaining proteins with an E-value threshold of 1 × 10^−5^. Finally, protein sequences were clustered into paralogous and orthologous genes using the program OrthoMCL, with a default inflation parameter for the Markov cluster algorithm.

To resolve the phylogenetic position of ‘Changxianggeng 1813’, the single-copy orthologous genes extracted from the above six *Oryza* genomes were aligned using MUSCLE v.3.8.3 [80] and then concatenated into a super-gene alignment matrix. Phylogenetic analysis was conducted using RAxML-HPC v.8.2.8 [81] with 1000 bootstrap replicates. The best model and parameter settings were chosen according to the Akaike Information Criterion (AIC) using jModelTest v.2.1.4 [82]. Divergence times between these six *Oryza* species/subspecies/cultivars were estimated by the program MCMCTree in PAML v.4.7 [83]. The following four divergence times obtained from the Timetree database (http://www.timetree.org/, accessed on 7 February 2019) were used for calibrations (in million years ago, Ma): (i) *O. barthii* and *O. sativa* (0.95–2.42 Ma), (ii) *O. nivara* and *O. sativa* (0.603–1.089 Ma), (iii) *O. rufipogon* and *O. nivara* (0.603–1.089 Ma), and (iv) *O. rufipogon* and *O. sativa* (0.598–1.255 Ma). To gain more insights into the evolutionary dynamics of the genes, the expansion and contraction of orthologous gene families were determined in these six members of *Oryza* with CAFÉ [84] and then subjected to GO functional annotation.

### 3.9. Analysis of Genomic Variations

MUMmer v.3.23 [85] was used to align the ‘Changxianggeng 1813’ (fragrant *japonica* cultivar) genome against the Nipponbare (non-fragrant *japonica* cultivar) genome by the nucmer utility under the parameters-mum. The delta-filter utility was subsequently used to filter repeats and determine the one-to-one alignment blocks in conjunction with parameters -1 -r -q. Single nucleotide polymorphisms (SNPs) and small insertion-deletion polymorphisms (InDels) were called from the filtered data using the show-snps function under the parameters -Clr TH.

Structural variants (SVs) were detected from the genome alignment between ‘Changxianggeng 1813’ and Nipponbare by using the show-diff function in MUMmer, and six SV types were obtained, including gap between two mutually consistent alignments (GAP), inserted duplication (DUP), other inserted sequence (BRK), rearrangement (JMP), rearrangement with inversion (INV), and rearrangement with another sequence (SEQ). The SVs with a minimum size of 1000 bp in length were retained in this study.

## 4. Conclusions

Here, we presented a high-quality reference genome sequence of a new fragrant rice cultivar ‘Changxianggeng 1813’, using a combination of Nanopore long reads, Illumina short reads, and Hi-C data. To our knowledge, this is the first de novo chromosome-level genome assembly for fragrant *japonica* rice. The ‘Changxianggeng 1813’ genome has a total length of ~378.78 Mb and comprises 31,671 high-quality protein-coding genes. Based on this annotated genome sequence, we demonstrated that it was the *badh2-E2* type of deletion (a 7 bp deletion in the second exon) that caused fragrance in this *japonica* rice cultivar. Through pairwise genome comparison between ‘Changxianggeng 1813’ and the non-fragrant *japonica* cultivar Nipponbare, a total of 289,970 SNPs, 96,093 InDels, and 8690 large SVs were identified. Undoubtedly, these genomic resources will promote the genic and genomic studies of rice and be beneficial for cultivar improvement of fragrant *japonica* rice. However, it should also be noted that our study has two notable limitations. First, we sequenced only a single individual, which was insufficient for investigating population genomic diversity, population structure, and cultivar origins of fragrant *japonica* rice. Second, our study still leaves a gap in our knowledge of genomic variations between *japonica*, *indica*, and *aus* type fragrant rice. Hence, we anticipate that further population-scale, long-read sequencing datasets, as well as improvements in genome comparison algorithms, will help overcome these limitations.

## Figures and Tables

**Table 1:** Statistics of the genome assembly of ‘Changxianggeng 1813’.

Genome Assembly	‘Changxianggeng 1813’
Total number of scaffolds	20
GC content (%)	43.55
Scaffold N50 (bp)	29,831,576
Scaffold N90 (bp)	16,183,542
Longest Scaffold	43,598,337
Total length (bp)	378,775,865
Mapping efficiency (%)	99.11%
Complete BUSCO (%)	96.16%

**Table 2:** Prediction of protein-coding gene models in the ‘Changxianggeng 1813’ genome.

Method	Software/Gene Set	Gene Number	Average Gene Length (bp)	Average CDS Length (bp)	Average Exon per Gene	Average Exon Length (bp)	Average Intron Length (bp)
De novo	Augustus	55,473	5539.04	2107.58	3.38	623.88	1442.88
	GlimmerHMM	35,280	2841.15	1183.67	4.81	246.27	435.45
Homolog	*Oryza brachyantha*	46,486	11,708.76	911.19	3.79	240.46	3871.05
	*Oryza sativa japonica*	55,308	10,634.66	964.91	3.6	268.35	3725.27
	*Aegilops tauschii*	55,839	13,039.16	939.32	3.55	264.97	4754.30
	*Panicum hallii*	48,315	11,231.61	911.68	3.66	249.38	3885.87
RNA-Seq	TransDecoder	12,400	4698.81	1203.90	6.73	329.13	433.89
Final set	EvidenceModeler	32,165	4243.68	1224.17	4.87	434.61	546.96

## Data Availability

The assembled genome of ‘Changxianggeng 1813’ and all raw sequencing data have been deposited under NCBI BioProject PRJNA856027 with accession nos. SRR20046019–SRR20046022.

## Supplementary Materials

The following supporting information can be downloaded at: https://www.mdpi.com/article/10.3390/ijms23179705/s1.
