# Supplementary material for: Chromosome-Level Genome Assembly of a Fragrant Japonica Rice Cultivar ‘Changxianggeng 1813’ Provides Insights into Genomic Variations between Fragrant and Non-Fragrant Japonica Rice
Source: Int J Mol Sci. 2022 Aug 26;23(17):9705. doi: 10.3390/ijms23179705 (PMC9456513; doi:10.3390/ijms23179705)
Supplement: Supplementary file 1 [file ijms-23-09705-s001.zip › Figure S1.pdf]

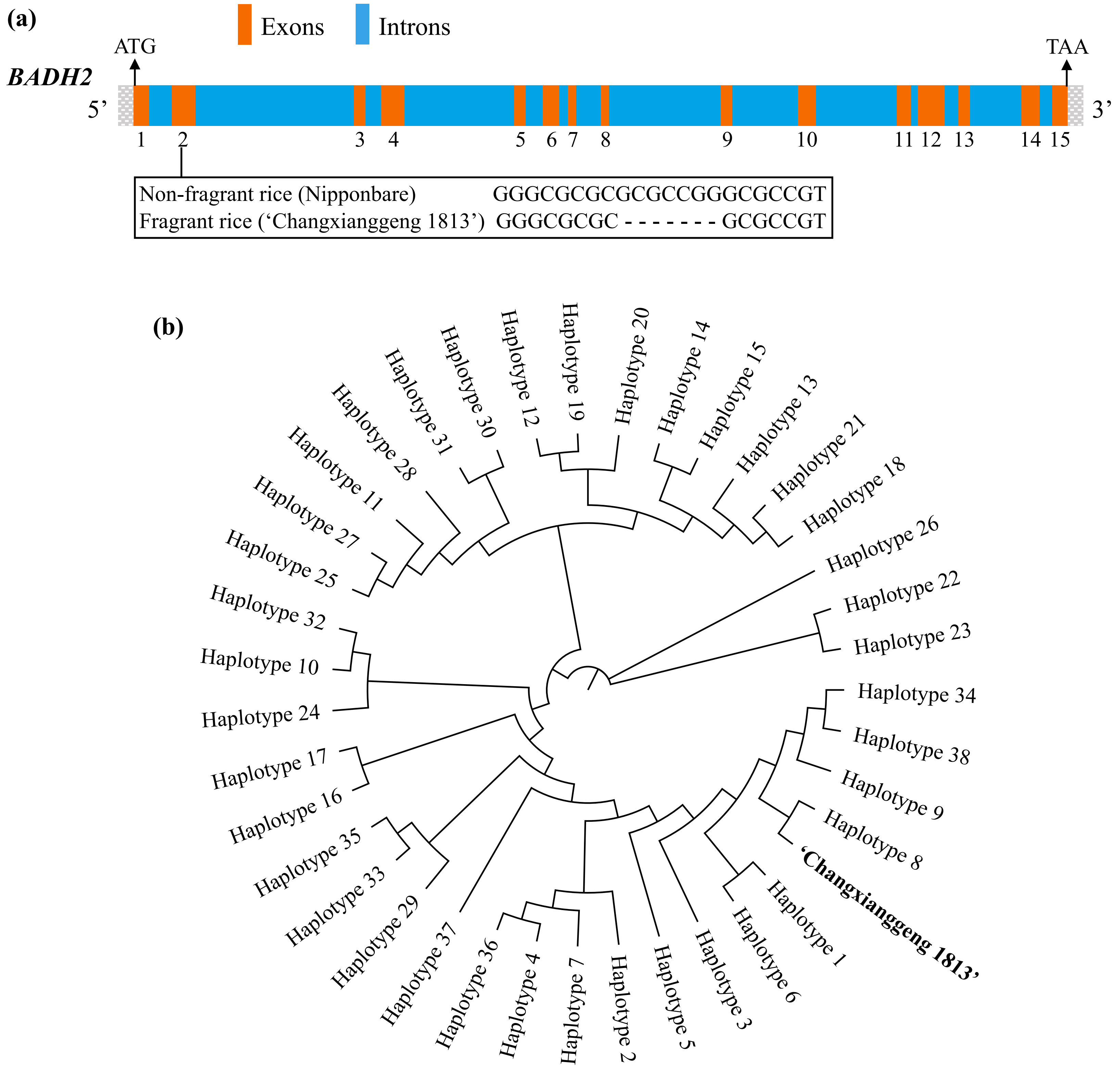

(a)
Exons
Introns
ATG
TAA
BADH2
5'
3'
1 2 3 4 5 6 7 8 9 10 11 12 13 14 15
Non-fragrant rice (Nipponbare) GGGCGCGCGCGCCGGGCGCCGT
Fragrant rice ('Changxianggeng 1813') GGGCGCGC - - - - - - - GCGCCGT
(b)
Haplotype 26
Haplotype 22
Haplotype 23
Haplotype 34
Haplotype 38
Haplotype 9
Haplotype 8
'Changxianggeng 1813'
Haplotype 1
Haplotype 6
Haplotype 3
Haplotype 5
Haplotype 2
Haplotype 7
Haplotype 4
Haplotype 36
Haplotype 37
Haplotype 29
Haplotype 33
Haplotype 35
Haplotype 16
Haplotype 17
Haplotype 24
Haplotype 10
Haplotype 32
Haplotype 25
Haplotype 27
Haplotype 11
Haplotype 28
Haplotype 31
Haplotype 30
Haplotype 12
Haplotype 19
Haplotype 20
Haplotype 14
Haplotype 15
Haplotype 13
Haplotype 21
Haplotype 18

Figure S1: (a) Structure of the BADH2 gene showing 5’ UTR, initiation codon (ATG), 15 exons, 14 introns, stop codon (ATT) and 3’ UTRs. The partial nucleotide sequence of second exon is shown for both non-fragrant (Nipponbare) and fragrant (‘Changxianggeng 1813’) rice. (b) Phylogenetic relationships among the BADH2 gene in ‘Changxianggeng 1813’ and 38 BADH2 haplotypes pre-ciously identified (see [2] for full details);
