# Supplementary material for: Chromosome-Level Genome Assembly of a Fragrant Japonica Rice Cultivar ‘Changxianggeng 1813’ Provides Insights into Genomic Variations between Fragrant and Non-Fragrant Japonica Rice
Source: Int J Mol Sci. 2022 Aug 26;23(17):9705. doi: 10.3390/ijms23179705 (PMC9456513; doi:10.3390/ijms23179705)
Supplement: Supplementary file 1 [file ijms-23-09705-s001.zip › Tables S1-S8.pdf]

Supplementary Tables

Table S1. The statistics of sequencing data used for the 'Changxianggeng 1813' genome assembly.

Libraries	Insert size (bp)	Clean data (Gb)	Mean read length (bp)	Sequence coverage (×)
Illumina	350 bp	28.21	150	74.48
Nanopore	–	51.59	10,760	136.20
Hi-C	350 bp	41.45	150	109.43
Total	–	121.15	–	320.11

Table S2. Estimation of genome size based on *19*-mer statistics.

***K*-mer**	***K*-mer_number**	***K*-mer_depth**	**genome size**	**heterozygosity rate**
19	18,243,910,499	45.78	394.39 bp	0.08%

Table S3. The length distribution of each chromosome.

Chromosome ID	Length (bp)
chr1	24,418,402
chr2	29,831,576
chr3	28,654,189
chr4	30,175,499
chr5	28,379,153
chr6	29,585,998
chr7	43,598,337
chr8	36,284,563
chr9	32,053,675
chr10	35,158,174
chr11	37,981,760
chr12	22,655,339

Table S4. Validation of the completeness of the genome assembly of 'Changxianggeng 1813' using the BUSCO approach.

Description	Number of genes	Percentage (%)
Complete BUSCOs	1,552	96.2
Complete and single-copy BUSCOs	1,514	93.8
Complete Duplicated BUSCOs	38	2.4
Fragmented BUSCOs	9	0.6
Missing BUSCOs	53	3.2

Table S5. Summary statistics of repeat sequences in the 'Changxianggeng 1813' genome.

Type	De novo + repbase		TE protiens		Combined TEs	
	Length (bp)	% in genome	Length (bp)	% in genome	Length (bp)	% in genome
DNA	92,052,249	24.3	8,063,604	2.13	92,687,141	24.47
LINE	6,484,115	1.71	3,153,876	0.83	6,741,267	1.78
SINE	447,867	0.12	0	0	447,867	0.12
LTR	91,097,914	24.05	26,154,923	6.91	91,493,064	24.15
Tandem repeats	3,661,198	0.97	0	0	3,661,198	0.97
Other	769	0	0	0	769	0
Unknown	3,742,890	0.99	7,938	0	3,750,828	0.99
Total	197,677,261	52.19	37,375,718	9.87	201,310,513	53.15

Table S6. Summary statistics of functional protein-coding genes in the 'Changxianggeng 1813' genome.

Database	Number	Percent (%)
Uniprot	31,309	97.34%
Pfam	25,333	78.76%
GO	23,775	73.92%
KEGG	10,869	33.79%
NR	29,105	90.49%
InterProScan	30,508	94.85%
All annotated	31,671	98.46%

Table S7. Summary statistics of non-coding RNAs in the 'Changxianggeng 1813' genome.

Type		Copy	Average length (bp)	Total length (bp)	% of genome
miRNA		1756	200	351273	0.092739
tRNA		715	75	53951	0.014243
rRNA	rRNA	322	167	53729	0.014185
	18S	8	1769	14153	0.003737
	28S	19	209	3980	0.001051
	5.8S	15	157	2358	0.000623
	5S	280	119	33238	0.008775
snRNA	snRNA	731	115	84116	0.022207
	CD-box	582	109	63189	0.016682
	HACA-box	64	130	8300	0.002191
	splicing	85	149	12627	0.003334

Table S8. Summary statistics of gene families among six *Oryza* species/subspecies/cultivars.

Accession	Gene number	Genes in families	Family number	Unique families	Average genes per family
'Changxianggeng 1813'	32165	32165	26658	1576	1.21
Nipponbare	24211	24211	21237	1370	1.14
O. rufipogon	47593	47593	34689	3148	1.37
O. sativa indica	37358	37358	29988	2267	1.25
O. nivara	48361	48361	34596	3384	1.4
O. barthii	41680	41680	31673	2517	1.32
